# Ubiquilin 1 suppresses the cancer stem cell–like traits of non-small cell lung cancer cells by regulating reactive oxygen species homeostasis

**DOI:** 10.1080/21655979.2021.1979353

**Published:** 2021-09-21

**Authors:** Ting Liu, Qianqian Ma, Wenjie Li, Yan Hu, Jun Yang, Qi Yao

**Affiliations:** Department of Geriatric Medicine, Ningbo First Hospital, Ningbo, China

**Keywords:** UBQLN1, cancer stem cell, reactive oxygen species, PGC1α, mitochondrial biogenesis

## Abstract

Cancer stem cell (CSC) has been confirmed to trigger tumor occurrence and progression and CSC can develop strategies to maintain a lower reactive oxygen species (ROS) level compared to cancer cells. However, the mechanisms contributing to ROS homeostasis in CSC are still lacking key elements. In the current study, we found that reductive redox states and ROS levels were suppressed in non-adherent spheres formed by non-small cell lung cancer (NSCLC) cells, which were confirmed to hold CSC-like traits. However, mitochondria DNA content and cellular oxygen consumption rate analyses revealed fewer numbers of mitochondria in NSCLC spheres. Further exploration attributed this result to decreased mitochondrial biogenesis, likely resulted from the accelerated degradation of peroxisome proliferator-activated receptor γ coactivator 1α (PGC1α). Mechanistic studies indicated that Ubiquilin 1 (UBQLN1) increased PGC1α protein stability via reducing the ubiquitination of PGC1α protein. Moreover, UBQLN1 was lowly expressed in NSCLC spheres compared to that in parental NSCLC cells and UBQLN1 overexpression suppressed the CSC-like traits of NSCLC cells, which was characterized as the decrease of ALDH1 activity, sphere-formation ability, and CSC marker expression. Finally, clinical investigations further demonstrated that UBQLN1 level was positively correlated with patient’s survival of lung adenocarcinoma, but not squamous cell carcinoma of lung. Taken together, our results revealed a novel mechanism involving ROS homeostasis and mitochondrial biogenesis in non-small cell lung CSCs, which may provide novel potential targets and methods for NSCLC patients.

## Introduction

The mortality rate of lung cancer ranks first among malignant tumors in the world and has become the first-killer of human health in malignant diseases, of which non-small cell lung cancer (NSCLC) accounts for about 85% [1]. Most NSCLC are in the late stage of recurrence or metastasis upon being clinically diagnosed. Plenty of evidences show that cancer stem cells (CSCs) are the ‘root’ of malignant tumor occurrence, progression, and recurrence [2]. In recent years, researchers have made in-depth and detailed observations on the biological characteristics of this subpopulation of cells, such as proliferation and differentiation, treatment resistance, invasion and metastasis ability, immune escape, hypoxia tolerance, and angiogenesis [3]. However, there is lacking relevant research on CSC mitochondria and related energy metabolism characteristics. Recent studies have shown that the morphology, distribution, quantity, and related energy metabolism parameters of mitochondria play an important role in regulating the self-renewal, pluripotency and proliferation/differentiation of normal stem cells [4]. Therefore, it will help us to deeply understand the biological characteristics of CSCs that observing the characteristics of mitochondria and related energy metabolism of CSCs. Meanwhile, in view of the core roles of CSCs in tumorigenesis, it will also help us to comprehensively understand the occurrence, development, and recurrence of malignant tumors.

Peroxisome proliferator activated receptor γ co-activator 1 (PGC-1) family, including PGC-1α, PGC-1β, and PGC-1 related coactivator (PRC), play a key role in regulating mitochondrial function and energy homeostasis, among which PGC-1α and PGC-1β have a sequence similarity in its whole length and have been widely linked with biological processes [5]. It was found that, in brown adipose tissue (BAT), PGC-1α could regulate the browning of white fat by increasing the expression of uncoupling protein (UCP) in mitochondria, which also plays an important role in adaptive heat generation [6]. Other studies have shown that changes of PGC-1α level are closely related to obesity, diabetes, lipid metabolism disorder, and cardiovascular diseases. But the circulating level of PGC-1α is affected by many factors, such as hypoxia, hypothermia, exercise, hunger, and insulin [7]. Additionally, PGC-1α overexpression can promote CD8 T cell adaptability, memory formation, and antitumor immunity [8]. Furthermore, studies have shown that adenosine monophosphate activated protein kinase (AMPK)/PGC-1α axis also plays a key role in regulating mitochondrial energy metabolism [9]. Notably, studies have shown that the purpose of treating a disease can be achieved by mediating PGC-1α-related pathway, upregulating or downregulating PGC-1α, or its upstream and downstream factors [10]. However, the upstream effectors of PGC-1α and its roles in non-small cell lung CSCs are still unknown.

Ubiquilin 1 (UBQLN1) encodes ubiquilin-1 protein (also known as PLIC-1; protein linking IAP [integrin-associated protein] with cytoskeleton-1), which belongs to a highly conserved group of ubiquitin-like proteins [11]. It is expressed in all tissues and tightly correlated with Alzheimer’s disease and cancer [12]. UBQLN1 contains ubiquitin like (UBL) region, ubiquitin related (UBA) region, and multiple heat shock chaperone (STI) regions [13]. UBQLN1 protein interacts with ubiquitinated protein through UBA region, and proteasome through UBL region. Therefore, it can act as a ‘bridge’ between ubiquitinated proteins and proteasomes, promote the degradation of ubiquitinated proteins, and thus participate in various pathological or physiological processes. Beverly et al. showed that UBQLN1 protein can interact with anti-apoptotic protein Bcl-b and promote the ubiquitination of multiple sites of Bcl-b, which is significant to maintain the stability of Bcl-b, exerting an important role in anti-apoptotic process [14]. Funakoshi et al reported that XDRP1, a homologous gene of UBQLN1 of Xenopus laevis, binds to cyclinA (A1 and A2) to prevent further differentiation of Xenopus embryonic cells. UBQLN1 can also interact with tumor suppressor proteins Dan and S protein to adjust DNA synthesis and then affect cell differentiation [15]. A few studies found that the level of UBQLN1 mRNA in lung adenocarcinoma was significantly higher than that in normal control. Chen et al. performed Western blot to compare 9 paired of lung adenocarcinoma and adjacent tissues, and found that the level of UBQLN1 protein in lung adenocarcinoma was higher than that in adjacent normal controls [16]. Serum UBQLN1 autoantibody played an important role in lung adenocarcinoma diagnosis [16]. These results suggest that UBQLN1 may be involved in the occurrence and development of lung adenocarcinoma, and UBQLN1 protein may have an important correlation with the clinicopathology of lung adenocarcinoma.

Here, non-adherent spheres, which were confirmed to hold CSC-like traits, were collected as non-small cell lung CSCs, and the reduction of redox states, ROS levels, mitochondria DNA content, and cellular oxygen consumption rate were found in non-adherent spheres. Further mechanistic studies revealed that PGC1α was recruited by UBQLN1, which subsequently regulated the ubiquitination level and stability of PGC1α protein. PGC1α increased mitochondrial biogenesis, this is responsible for UBQLN1-mediated suppression on the CSC-like traits of NSCLC cells. Together, our results identified UBQLN1 as a key modulator of non-small cell lung CSC progression and demonstrated a novel UBQLN1/PGC1α axis involving ROS homeostasis and mitochondrial biogenesis in non-small cell lung CSCs.

## Materials and methods

### Cell culture

NSCLC cell lines A549 and H1299 were purchased from Procell Life Science&Technology Co.,Ltd (Wuhan, China). BEAS2B and 293 T cells were cultured in DMEM medium (Cat # KGM12800-500, Keygen, Nanjing, China) and lung cancer cells were cultured in RPMI1640 medium (Cat # KGM31800-500, Keygen) in 37°C and 5% CO_2_, both of which were added with 1% streptomycin (Cat # A100382, Sangon, Shanghai, China) and penicillin (Cat # A100339, Sangon, Shanghai, China).

### Plasmid construction, siRNA synthesis, and transfection

UBQLN1 and PGC1α coding sequences were inserted into pcDNA4.1 vector to obtain the overexpression vectors (pc-UBQLN1 and pc-PGC1α), and empty vector was served as a control. SiRNAs against UBQLN1 (Cat # CRH9311) and PGC1α (Cat # CRH7421) were purchased from Cohesion Biosciences (Suzhou, Jiangsu, China), and corresponding negative control (NC) was used as a control. The transfection procedure was constructed using Jetprime (Cat # PT-114-15, Polyplus Transfection, Illkirch, France) according to the recommending protocols.

### Sphere-formation analysis

Sphere formation analysis was performed to analyze the CSC-like traits of NSCLC cells and collect NSCLC spheres. Briefly, cells were cultured in 37°C, 5% CO_2_ incubator, low-adherent culture plates (Cat # 174,930, Thermo Fisher Scientific, Waltham, MA, USA) with sphere-culturing medium containing DMEM/F12 (Cat # 31,331,093, Thermo Fisher Scientific, Waltham, MA, USA) with 1% methylcellulose (Cat # M0512, Sigma) and 10 ng/ml FGF-β (Cat # 11,343,623, ImmunoTools), 10 ng/ml EGF (Cat # 11,343,406, ImmunoTools) and 1 × B27 (Cat # 17,504,044, Thermo Fisher Scientific). Ten days later, sphere size and number were observed under microscope. For experiments of spheres, spheres were collected, disaggregated, re-seeded into plates followed by the protocols of cell culture, and subjected to further experiments, or the disaggregated spheres were subjected to sphere-formation analysis again.

### ALDH1 activity detection

ALDH1 activity detection kit (Cat # MAK082, Sigma) was used to determine the ALDH1 activity according to the recommended procedure from manufacturers.

### ROS detection

ROS Assay Kit (Cat # 50101ES01, YEASEN, Shanghai, China) was used to detect ROS level. Briefly, before loading the probe, DCFH-DA was diluted with serum-free medium according to 1:1000 to make its final concentration 10 μM. Then cells were collected by centrifugation, and properly diluted probes were added to make the cell density 1.0 × 10^6^/mL and wash the cells with serum-free cell culture medium for 2 times to fully remove DCFH-DA that does not enter the cells. ROS level was subsequently detected using flow cytometry.

### Mitochondrial membrane potential (MMP) detection

Mitochondrial Permeability Transition Pore Assay Kit (flow cytometry) (Cat # 40756ES60, YEASEN) was used to determine MMP level. Take out the reagent and return it to room temperature, 50 µL DMSO was used dissolve 1 tube of Calcein AM, mix well and store it away from light. Cells were prepared into a single cell suspension with HBSS/Ca to the density of 1 × 10^6^ cells/ml. Divide each sample into three parts. Subsequent staining sequence: tube 1 only contains Calcein, tube 2 contains Calcein and CoCl_2_, tube 3 contains Calcein, CoCl_2_ and ionomycin, and another tube of cell sample without reagent is prepared for corresponding instrument settings. Dilute 1 mM Calcein AM with HBSS/Ca at 1:500 dilution ratio to prepare 2 µM working solution. Add 5 µL Calcein AM working solution into the 3 tubes, respectively, and mix well. Add 5 µL CoCl_2_ into tubes 2 and 3, and mix well. Add 5 µL lionomycin (100 µM) to tube 3 and mix well. Incubate at 37°C in dark for 15 min. Then 3.5 mL HBSS/Ca was added to each tube and the cells were collected by centrifugation. Re-suspend the cell suspension with 400 µL of suitable buffer that can also be used for flow cytometry. Subsequently, samples were placed on ice and analyzed by flow cytometry within 1 h. Set up the corresponding instrument with cell sample without reagent. Fluorescence analysis was carried out with a flow 488 exciter. The maximum excitation wavelength of Calcein AM was 494 nm and the maximum emission wavelength was 517 nm.

### Analysis of mitochondrial DNA (mtDNA) level

MtDNA level was detected using Mitochondrial DNA (mtDNA) nucleic acid detection kit (PCR fluorescent probe method) (Cat # SA-5041, Beijing, China) following the standard protocols.

### Oxygen consumption rate (OCR) determination

MitoCheck Mitochondrial OCR Assay Kit (Cat # 600,800, Cayman Chemical, Ann Arbor, MI, USA) was used to measure OCR following the procedure mentioned in the previous study [17].

### Redox ratio evaluation

NAD/NADH, GSH/GSSG, and NADPH/NADP ratio was measured using NAD+/NADH Assay Kit with WST-8 (Cat # S0175, Beyotime, China), GSH and GSSG Assay Kit (Cat # S0053, Beyotime), and NADP+/NADPH Assay Kit with WST-8 (Cat # S0179, Beyotime), respectively, according to the manufacturer’s protocol.

### Western blot, protein ubiquitination, and stability analysis

Cells were digested with 0.05% trypsin and 0.02% EDTA for 30 s – 2 min. Then complete medium was added to terminate the digestion. The supernatant was centrifuged at 4°C 800 r/min, and added 200 μL 1% SDS protease inhibitor to lyse cells on ice bath. The protein concentration was quantified following the instructions of Pierce protein assay kit. Before use, freeze-thaw samples on ice, take a certain volume (including 50 μg protein) into a clean Eppendorf tube and add 4 μL 5 × SDS loading buffer, 1% SDS to 20 μL. Denatured at 95°C for 5 min, the samples were placed on ice and loaded as soon as possible. 10% SDS-PAGEs were used to separate the proteins, which were then transferred to the ECL membranes (100 V, 1 h), sealed with 10% no-fat milk for 2 h at 5% PBST, and then incubated with primary antibodies overnight at 4°C. PBST solution was used to wash membranes three times, which were then reacted with horseradish peroxidase labeled Goat anti mouse Ig at room temperature for 1.5 h. Finally, membranes were exposed using an ECL kit (Cat # E411-03, Vazyme, Nanjing, China) to detect protein expression. GAPDH served as an endogenous control. For protein ubiquitination assay, MG132 was added into cells 6 h before collecting the samples. For protein stability assay, cycloheximide (CHX) (Cat # 40325ES03, YEASEN, Shanghai, China) was used to inhibit protein synthesis.

### Real time quantitative PCR (RT-qPCR)

1 mL Trizol reagent (Cat # R401-01, Vazyme, Nanjing, China) was used to extract total RNA of 10^6^ cells. The absorbance value of A_260_ and A_280_ of RNA should be 1.8≤ A_260_/A_280_ ≤ 2.0, and the RNA concentration should be calculated. M-MLV reverse transcriptase (Cat # R021-01, Vazyme) was used to reversely transcribe RNA at 37°C for 1 h in 10 μL system and inactivate reverse transcriptase at 95°C for 3 min. the synthesized cDNA was saved at −20°C. 20 μL reaction system containing 2 × Quantitative PCR buffer (Cat # Q711-02, Vazyme) 10 μL, 2 μL upstream and downstream primers was used to detect the relative expression levels of transcripts. The reaction conditions were as follows: pre-denaturation at 93°C for 3 min, 93°C for 30 s, 56°C for 40 s. GAPDH was served as an endogenous control.

### Analysis of the correlation between gene expression and survival of lung cancer patients

Kaplan – Meier (KM) Plotter online tool was utilized to analyze the relationship between the expression of UBQLN1 and overall survival, and post-progression survival of lung cancer patients (including lung adenocarcinoma patients and squamous cell carcinoma of lung) following the concrete parameters [18].

## Statistical analysis

Graphpad Prism 8.0 statistical software was used to analyze the significance between groups. The measurement data are expressed as χ ± s. T test was used to compare the two groups. P < 0.05 was statistically significant.

## Results

### Decreased ROS levels in NSCLC spheres compared to that in NSCLC cells

**Figure 1.**
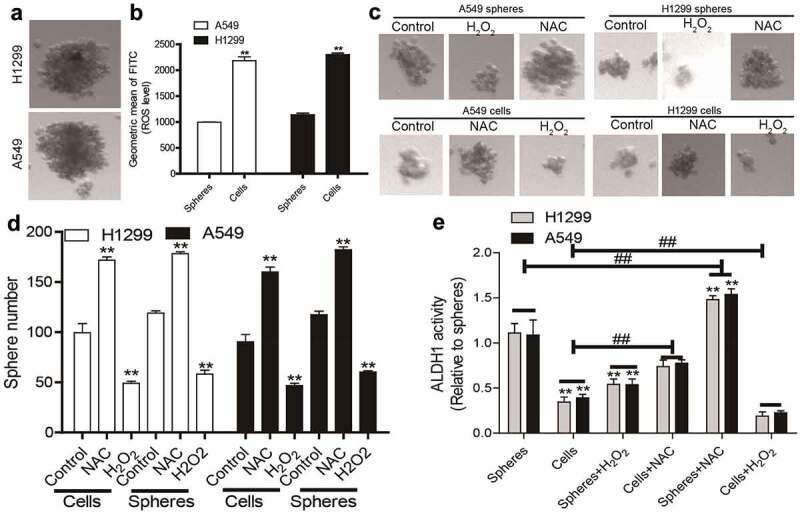
**Decreased ROS levels in NSCLC spheres compared to that in NSCLC cells**. (a) Images of spheres formed by NSCLC cells. (b) ROS level was measured in NSCLC spheres and parental cells. (c) Sphere size was evaluated in NSCLC spheres and cells with or without H_2_O_2_, and NAC treatment. (d) Sphere number was examined in NSCLC spheres and cells with or without H_2_O_2_, and NAC treatment. (e) ALDH1 activity was determined in NSCLC spheres and cells with or without H_2_O_2_, and NAC treatment. n ≥ 3, **P < 0.01 vs. Control, ^##^P < 0.01 vs. Cells

To mimic the characteristics of CSC, non-adherent spheres, which have been firmly confirmed to hold CSC-like traits, were collected in NSCLC cells (Figure 1a). It was found that NSCLC spheres exhibited a lower ROS level compared to that in parental NSCLC cells (Figure 1b). Additionally, increasing ROS level using hydrogen peroxide (H_2_O_2_, a ROS inducer) attenuated the sphere-formation ability in both NSCLC spheres and cells, while the ROS scavenger N-acetyl-cysteine (NAC) enhanced it, as evident by the change of sphere number and size (Figure 1c and 1d). Furthermore, ALDH1 activity was increased in NSCLC spheres relative to that in NSCLC cells, which was reduced and upregulated by hydrogen peroxide and NAC, respectively (Figure 1e). Thus, a low ROS level might be a critical factor for maintaining the CSC-like traits of NSCLC cells.

### NSCLC spheres retain less respiratory capacity and mitochondrial content

**Figure 2.**
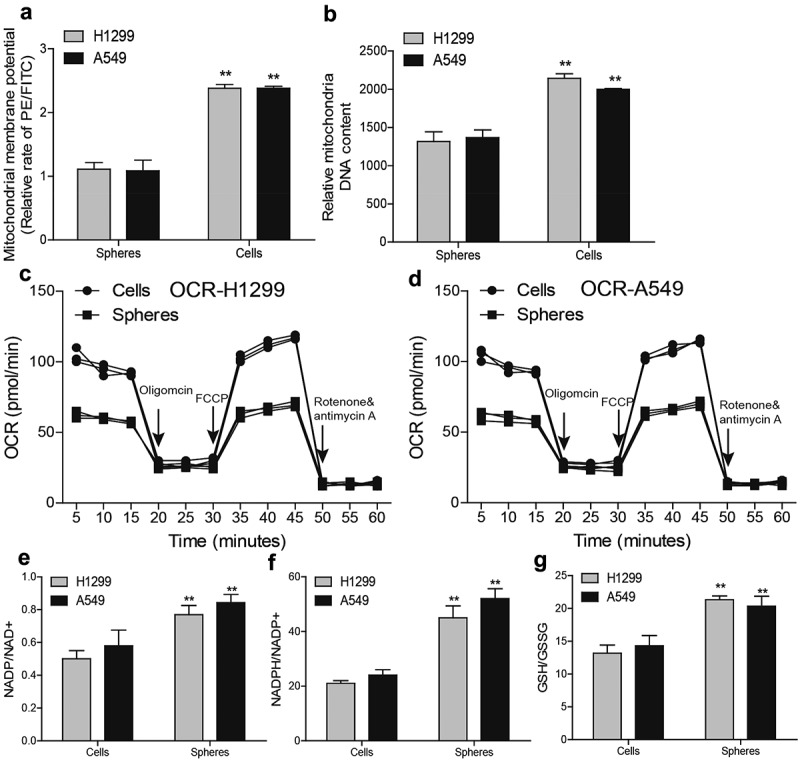
**NSCLC spheres retain less respiratory capacity and mitochondrial content**. (a) MMP level was examined in NSCLC spheres and cells. (b) MtDNA content was evaluated in NSCLC spheres and cells. (c) OCR was determined in H1299 spheres and cells. (d) OCR was measured in A549 spheres and cells. (e) NADP/NAD+ ratio was detected in NSCLC spheres and cells. (f) NADPH/NADP+ ratio was examined in NSCLC spheres and cells. (g) GSH/GSSG ratio was evaluated in NSCLC spheres and cells. n ≥ 3, **P < 0.01 vs. Control

To delineate the underlying mechanisms of discrepancies in ROS between NSCLC spheres and cells, we focused on mitochondria roles as mitochondria are the main organelles exerting ROS in cells. We firstly detected mitochondrial membrane potential (MMP) through flow cytometry and found that MMP level was lower in NSCLC spheres than that in NSCLC cells (Figure 2a). Additionally, the mitochondrial DNA (mtDNA) level was lower in NSCLC spheres than in parental cells (Figure 2b). Furthermore, NSCLC spheres exhibited a lower level of the oxygen consumption rate (OCR) than that of NSCLC cells (Figure 2c and 2d). Moreover, the redox balance between biological redox couples was monitored in NSCLC spheres and parental cells, such as NAD/NADH, GSH/GSSG, and NADPH/NADP. As shown in Figure 2e – 2g, a relative reductive state was identified in NSCLC spheres compared with parental cells. Therefore, these results demonstrate that non-small cell lung CSCs retain a better mitochondrial integrity and function with less respiratory capacity and mitochondrial content, reducing ROS production.

### ROS generation and mitochondrial biogenesis are suppressed by the acceleration of PGC1α degradation in NSCLC spheres

**Figure 3.**
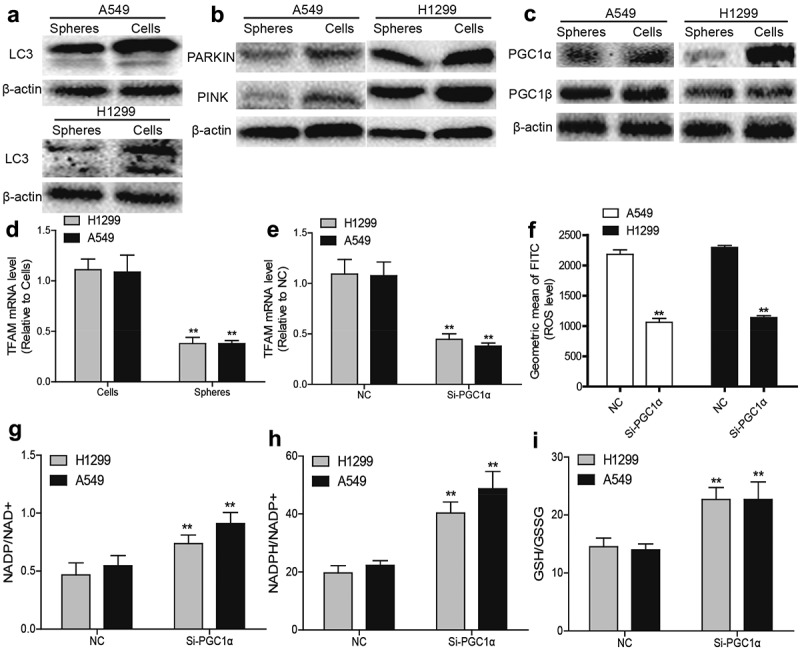
**ROS generation and mitochondrial biogenesis are suppressed by the acceleration of PGC1α degradation in NSCLC spheres**. (a) LC3 protein level was determined in NSCLC spheres and cells. (b) PARKIN and PINK protein levels were detected in NSCLC spheres and cells. (c) PGC1α and PGC1β protein levels were examined in NSCLC spheres and cells. (d) TFAM mRNA level was measured in NSCLC spheres and cells. (e) TFAM mRNA level was detected in NSCLC cells with or without PGC1α knockdown. (f) ROS level was examined in NSCLC cells with or without PGC1α knockdown. (g) NADP/NAD+ ratio was detected in NSCLC cells with or without PGC1α knockdown. (h) NADPH/NADP+ ratio was examined in NSCLC cells with or without PGC1α knockdown. (i) GSH/GSSG ratio was evaluated in NSCLC cells with or without PGC1α knockdown. n ≥ 3, **P < 0.01 vs. Control

To reveal the mechanisms underlying the reduced mitochondrial content in NSCLC spheres, mitochondria degradation was evaluated. As mitophagy is a main mechanism for reducing ROS stress and mitochondria degradation, mitophagy levels were measured in NSCLC spheres and parental cells. We found a lower LC3II (reflecting autophagic activity) level in NSCLC spheres than parental cells (Figure 3a). Additionally, PARKIN and PINK (mitophagy-specific markers) levels were reduced in NSCLC spheres (Figure 3b). Then mitochondrial biogenesis was evaluated in NSCLC spheres and parental cells. As the PGC1 family of transcriptional coactivators such as PGC1α and PGC1β, are master regulators modulating mitochondrial transcription factor A (TFAM) to maintain mitochondrial function and mtDNA levels, we detected PGC1α and PGC1β protein levels in NSCLC spheres and cells. As shown in Figure 3c, PGC1α was lowly expressed in NSCLC spheres, while PGC1β displayed a little change. Consistently, TFAM mRNA levels were downregulated in NSCLC spheres compared to that in NSCLC cells (Figure 3d). Furthermore, knockdown of PGC1α downregulated TFAM mRNA level (Figure 3e) and ROS generation in NSCLC cells (figure 3f). Moreover, knockdown of PGC1α resulted in a reductive state in NSCLC cells (Figure 3g – 3i). Together, these results suggest that the decreased PGC1α in NSCLC spheres might contribute to the reduction of mitochondrial biogenesis and ROS generation.

### UBQLN1 suppresses PGC1α degradation in NSCLC cells

**Figure 4.**
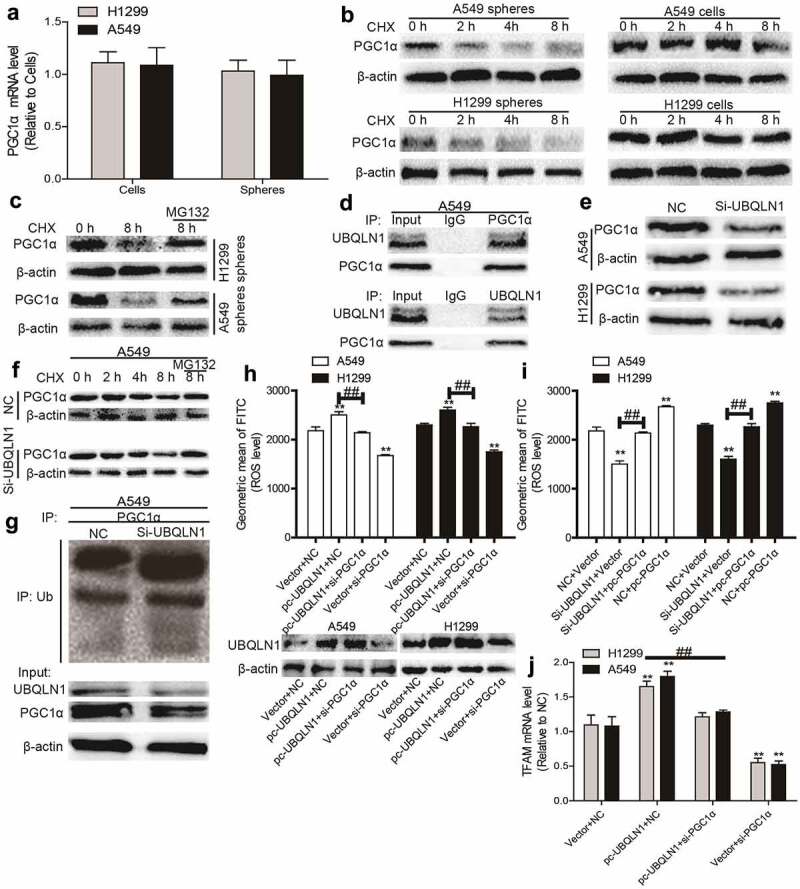
**UBQLN1 suppresses PGC1α degradation in NSCLC cells**. (a) PGC1α mRNA level was evaluated in NSCLC spheres and cells. (b) PGC1α protein stability was determined in NSCLC spheres and cells. (c) PGC1α protein was measured in NSCLC spheres treated with CHX as well as MG132 or not. (d) Co-IP analysis was performed to test the UBQLN1-PGC1α interaction. (e) PGC1α protein level was examined in NSCLC cells with or without UBQLN1 knockdown. (f) PGC1α protein was measured in A549 cells treated with CHX as well as MG132 or not. (g) PGC1α protein ubquitination level was detected in A549 cells with or without UBQLN1 knockdown. (h) ROS level was measured in NSCLC cells with UBQLN1 overexpression as well as PGC1α knockdown or not. (i) ROS level was examined in NSCLC cells with UBQLN1 knockdown as well as PGC1α overexpression or not. (j) TFAM mRNA level was determined in NSCLC cells with UBQLN1 overexpression as well as PGC1α knockdown or not. n ≥ 3, **P < 0.01 vs. Control, ^##^P < 0.01 vs. pc-UBQLN1+ NC or Si-UBQLN1+ Vector

We then explored the upstream regulators of PGC1α in NSCLC. It was found that the mRNA level of PGC1α was unchanged between NSCLC spheres and cells although its protein level was reduced in NSCLC spheres (Figure 4a), which means that PGC1α was post-transcriptionally regulated in NSCLC. Then PGC1α protein stability was analyzed in NSCLC spheres and cells treated with CHX (an inhibitor of protein synthesis) and an accelerated degradation of PGC1α was observed in NSCLC spheres (Figure 4b). Additionally, PGC1α protein was increased in NSCLC spheres treated with MG132 (blocking the proteasome) (Figure 4c). These results suggest that PGC1α protein degradation was increased in NSCLC spheres.

We continued to dissect the underlying mechanisms of accelerated degradation of PGC1α protein in NSCLC spheres. Since protein ubiquitination is critical for protein degradation and can be regulated by ubiquilins, and the previous studies have shown that UBQLN1 could mediate PGC1β protein degradation, we wondered whether PGC1α protein stability could be regulated by UBQLN1. As shown in Figure 4d, Co-IP experiments demonstrated that UBQLN1 interacted with PGC1α. Furthermore, knockdown of UBQLN1 decreased PGC1α protein expression (Figure 4e) and promoted the degradation of PGC1α protein in NSCLC cells, which could be rescued by MG132 treatment (figure 4f). Importantly, knockdown of UBQLN1 increased the ubiquitination level of PGC1α protein (Figure 4g). Moreover, knockdown of UBQLN1 resulted in decreased ROS level, which was partially rescued by PGC1α overexpression (Figure 4h). Further assays demonstrated that knockdown of PGC1α partially rescued UBQLN1-induced increase of ROS (Figure 4i) and TFAM mRNA level (Figure 4j) in NSCLC spheres. These results suggest that UBQLN1 decreased the ubiquitination level of PGC1α protein and thus blocked PGC1α protein degradation, which is necessary for the upregulation of mitochondrial biogenesis and ROS generation.

### UBQLN1 is lowly expressed in NSCLC spheres and inhibits the CSC-like traits of NSCLC cells dependent on PGC1α

**Figure 5.**
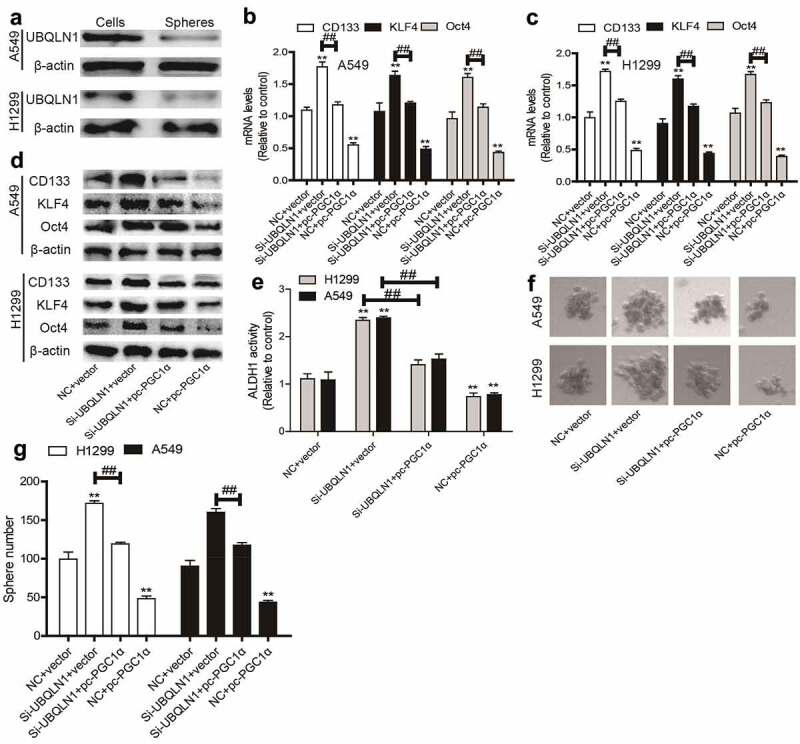
**UBQLN1 is lowly expressed in NSCLC spheres and inhibits the CSC-like traits of NSCLC cells dependent on PGC1α**. (a) UBQLN1 protein was detected in NSCLC spheres and cells. (b and c) CSC markers’ mRNA levels were measured in NSCLC cells with UBQLN1 knockdown as well as PGC1α overexpression or not. (d) CSC marker protein levels were determined in NSCLC cells with UBQLN1 knockdown as well as PGC1α overexpression or not. (e) ALDH1 activity was evaluated in NSCLC cells with UBQLN1 knockdown as well as PGC1α overexpression or not. (f and g) Sphere size and number were measured in NSCLC cells with UBQLN1 knockdown as well as PGC1α overexpression or not. n ≥ 3, **P < 0.01 vs. Control, ^##^P < 0.01 vs. Si-UBQLN1+ Vector

Then we wondered whether UBQLN1 could suppress the CSC-like traits of NSCLC cells. As expected, UBQLN1 protein expression was inhibited in NSCLC spheres compared with that in NSCLC cells (Figure 5a). UBQLN1 was then knocked down in NSCLC cells and we found that UBQLN1 knockdown increased the expression of CSC markers (CD133, KLF4, Oct4) (Figure 5b – 5d), ALDH1 activity (Figure 5e), and sphere-formation ability (figure 5f – 5g), which were rescued by PGC1α overexpression. Thus, our results indicate that UBQLN1 suppresses the CSC-like traits of NSCLC cells through PGC1α.

### UBQLN1 predicts a longer survival in lung adenocarcinoma patients, but not in squamous cell carcinoma of lung patients

**Figure 6.**
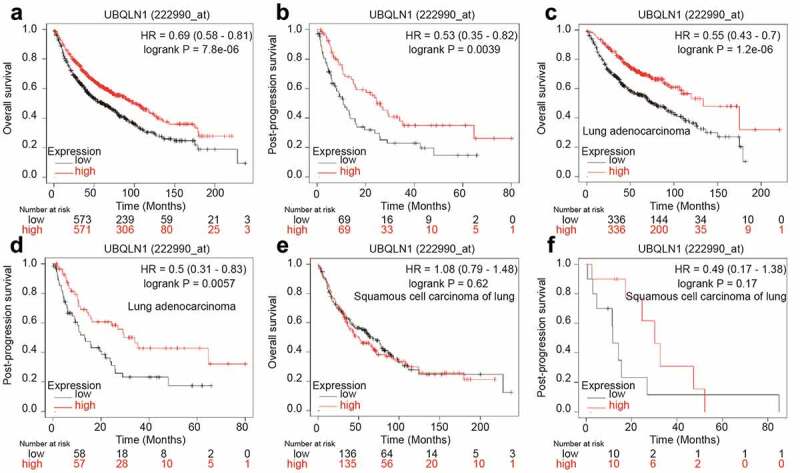
**UBQLN1 predicts a longer survival in lung adenocarcinoma patients, but not squamous cell carcinoma of lung patients**. (a) The correlation between UBQLN1 mRNA level and overall survival of lung cancer patients was evaluated through online dataset analysis. (b) The correlation between UBQLN1 mRNA level and post-progression survival of lung cancer patients was analyzed through online dataset analysis. (c and d) The correlation between UBQLN1 mRNA level and overall survival, post-progression survival of lung adenocarcinoma cancer patients was examined through online dataset analysis. (e and f) The correlation between UBQLN1 mRNA level and overall survival, post-progression survival of squamous cell carcinoma of lung patients was examined through online dataset analysis

Finally, we analyzed the correlation between UBQLN1 expression and the survival of lung cancer patients through the online dataset. As shown in Figure 6a and 6b, UBQLN1 expression was positively correlated with overall survival (OS) and post-progression survival (PPS) of lung cancer patients. However, when patients were split into lung adenocarcinoma patients and squamous cell carcinoma of lung, UBQLN1 expression was just positively correlated with the OS and PPS of lung adenocarcinoma patients (Figure 6c and 6d), but not with squamous cell carcinoma of lung patients (Figure 6e and 6f). These results demonstrate that UBQLN1 might function as a tumor suppressor only in lung adenocarcinoma patients.

## Discussion

Many methods have been used to treat NSCLC; however, treatment resistance, recurrence, and metastasis always happen and become the leading reasons of patient death [19]. CSC theory holds that CSCs are the seeds resulting in treatment tolerance, recurrence, and metastasis; however, there are no drugs targeting CSCs for clinical application, which might be due to the unclear mechanisms contributing to CSC progression [20]. Here, we focused on the underlying mechanisms contributing to non-small cell lung CSC progression as NSCLC is the first-killer of human health in malignant diseases. Although numerous studies have indicated the possible mechanisms for non-small cell lung CSC progression, in which the roles of redox homeostasis and metabolic reprogramming remain to be clarified.

Solid tumors are considered to be in hypoxic environment, while the hypoxic characteristics of CSCs are more obvious [21]. Studies have shown that ROS could exert multi-faced effects on tumor progression and the hence of that on CSC progression [22]. ROS can promote or suppress tumor progression in the early stage or late stage, respectively. During drug treatment, such as chemotherapy, many drugs can exert excessive ROS to kill cancer cells; however, CSCs can develop strategies to overcome the excessive production of ROS, thus acquiring resistance [21]. Currently, we also showed that ROS generation was significantly lower in NSCLC spheres compared to that in parental NSCLC cells. Mitochondria are the main location of producing ROS and maintaining redox homeostasis, this is the main factor regulating the increased ROS level induced by hypoxic environment [23]. Reduction of mitochondrial biogenesis often reduces ROS level, subsequently provides a reduced condition for cells and makes cell more adaptable for drug treatment. Here, it was demonstrated that NSCLC spheres exhibited a decreased ROS generation and mitochondrial biogenesis, hinting an important role of redox metabolism and reprogrammed ROS in non-small cell lung CSCs. Consistently, a previous report found that phosphoglycerate dehydrogenase (PHGDH) is an important contributor for drug resistance in hepatocellular carcinoma [24]. And inactivating PHGDH can promote ROS production and thus trigger apoptosis of hepatocellular carcinoma cells by drug treatment, like sorafenib [24]. In addition, a lower mitochondrial volume is tightly involved in the Warburg effect, which also has been shown to facilitate tumor progression and be an important metabolic trait maintaining CSC stemness [25]. A recent study demonstrated that overexpression of mitochondrial fission factor, which is significantly upregulated in liver CSCs, enhanced the stemness and tumor-initiating capability of liver CSCs [26]; Mitochondrial fission factor FIS1 can promote the stemness of lung cancer cells by regulating mitophagy; And suppressing the IFNGR-JAK-STAT-PARP1 pathway can attenuate the CSC-like traits of hepatocellular carcinoma via inducing mitochondrial alteration and DNA damage [27].

PGC1 transcriptional family, including PGC1α, PGC1β, and PGC1-related coactivator, regulates mitochondrial biogenesis [5]. These proteins can interact with other transcription factors engaging in mitochondrial gene expression to regulate the transcription of the mitochondrial genome, such as TFAM [5]. PGC1α and PGC1β act as co-transcriptional factors to enhance mitochondrial biogenesis [28]. However, their effects in cancer progression are not completely elucidated. PGC1α has been confirmed to acts as a metabolic modulator in cancer [29], and it can suppress melanoma metastasis [30]. Recently, it has been shown that the CSC-like traits of pancreatic precursor lesions can be driven by PGC1α-mediated metabolic reprogramming [31]. Other studies indicated that the ubiquitin–proteasome pathway is engaged in regulating PGC1α protein expression. However, the detailed upstream effectors are not fully revealed. In this study, enhanced ubiquitin-mediated proteolysis of PGC1α was identified in NSCLC spheres, resulting in the reduction of ROS generation and mitochondrial biogenesis, during which UBQLN1 played a critical role. However, although PGC1β protein was identified to hold the similar mechanisms in sorafenib-resistant hepatocellular carcinoma cells [32], it was not found in NSCLC spheres, which means the cell-specific character of PGC1α/β ubiquitination. Thus, further investigations should focus on the target-dependent functions of UBQLN1.

## Conclusion

All in all, our study demonstrated an UBQLN1-PGC1α axis-mediated ROS homeostasis and mitochondrial biogenesis which plays an important role in non-small cell lung CSC progression, providing a potential mechanism for NSCLC treatment.

## Supplementary Material

Supplemental Material

## Data Availability

All data generated or analyzed during this study are included in this published article.
