# Supplementary material for: Ubiquilin 1 suppresses the cancer stem cell–like traits of non-small cell lung cancer cells by regulating reactive oxygen species homeostasis
Source: Bioengineered. 2021 Sep 21;12(1):7132–44. doi: 10.1080/21655979.2021.1979353 (PMC8806721; doi:10.1080/21655979.2021.1979353)
Supplement: Supplemental Material [file KBIE_A_1979353_SM4632.doc]

CSC can develop strategies to maintain a lower reactive oxygen species (ROS) level compared to cancer cells. However, the mechanisms contributing to ROS homeostasis in CSC are still lacking key elements. Here, we examined the role of UBQLN1 in NSCLC stemness. UBQLN1 increased PGC1α protein stability via reducing the ubiquitination of PGC1α protein, and thus increased mitochondrial biogenesis, which decreased ROS production, resulting in the reduction of NSCLC stemness.
